# Feasibility of Partnering with Emergency Medical Services to Identify People at Risk for Uncontrolled High Blood Pressure

**Published:** 2012-02-02

**Authors:** Hendrika Meischke, Carol Fahrenbruch, Brooke Ike, Peggy Hannon, Jeffrey R. Harris

**Affiliations:** Department of Health Services, University of Washington; Seattle & King County Division of Emergency Medical Services, Seattle, Washington; Department of Health Services, University of Washington, Seattle, Washington; Department of Health Services, University of Washington, Seattle, Washington; Department of Health Services, University of Washington, Seattle, Washington

## Abstract

**Introduction:**

Uncontrolled high blood pressure (HBP) is a significant health problem and often goes undetected. In the prehospital care-delivery system of 9-1-1 emergency medical services (EMS) calls, emergency medical technicians (EMTs) routinely collect medical information, including blood pressure values, that may indicate the presence of chronic disease. This information is usually archived without any further follow-up. We conducted several planning activities during the fall of 2006 to determine if a partnership between researchers at the Health Marketing Research Center at the University of Washington, Public Health Seattle King County EMS division, and several large fire departments could be developed to help identify community residents with uncontrolled HBP and determine the most effective way to communicate HBP information to them.

**Methods:**

We partnered with 4 King County, Washington, fire departments that provide 9-1-1 EMS to develop an intervention for people with uncontrolled HBP who were attended by EMTs in response to a 9-1-1 call for assistance. On the basis of discussions with EMS personnel at all levels, we developed a system by which we could identify at-risk community residents by using medical incident report forms that EMS personnel completed; we consulted with EMS personnel to determine the most effective means of reaching these people. In addition we developed a survey to assess community residents' beliefs about blood pressure control, the role of EMTs as health care providers, and the convenience of fire stations as places to have blood pressure checked. Using contact information that EMS personnel obtained, we surveyed 282 community residents from a total of 794 people whom EMTs had identified as at risk for uncontrolled HBP to help us understand our target audience.

**Results:**

In consultation with EMS personnel, we determined that direct mail was the most effective way to reach people with uncontrolled HBP identified from EMS records to advise them of their risk. On the basis of the number with a known response to each question, 67% (n = 180/269) of the respondents reported that a doctor or other health professional had told them they had HBP, 95% (246/259) believed that regular screening for HBP was important, 65% (166/254) said that EMTs were highly credible health care providers, and 82% (136/165) said that they would feel comfortable receiving blood pressure screening at a local fire station.

**Conclusion:**

Partnering with local EMS may be an effective way to identify and reach community residents with uncontrolled HBP with information on their medical condition and to encourage them to have follow-up screening.

## Introduction

High blood pressure (HBP) is a risk factor for heart attack and stroke (1). In the United States, heart disease is the leading cause of death, and stroke is the third. HBP affects approximately 74 million Americans (1). Randomized controlled trials have shown that lowering blood pressure significantly reduces cardiovascular disease (2). Guidelines recommend target systolic blood pressure at or below 140 mm Hg and diastolic blood pressure at or below 90 mm Hg (1,3).

Because blood pressure screening is an inexpensive and effective diagnostic method, the National Commission on Prevention Priorities has listed it as a priority clinical preventive service based on their criteria of clinical burden and cost-effectiveness (4). Nonetheless, HBP is often inadequately controlled (5-7).

Participation in screening for a disease is related, in part, to a person's perceived risk of the disease and the benefits of recommended action (8). The Health Belief Model is a value-expectancy model that has been applied to many health behaviors, particularly screening behavior (9,10). According to the model, a cue to action triggers cognitive deliberations about the perceived threat of a disease and about barriers to and benefits of the recommended behavior, which influence a person's likelihood of taking a particular action. Interventions to increase screening for HBP could benefit from incorporating constructs of the Health Belief Model; we decided to base the content of the intervention on these principles by emphasizing that the person's blood pressure was high during a recent 9-1-1 visit (perceived susceptibility), that uncontrolled hypertension is very dangerous (perceived severity), and that there are treatments available to control HBP (perceived benefits). Because one of the barriers to screening is having a source of regular medical care, we wanted to encourage people to come to a local fire station for a free blood pressure check.

Measuring blood pressure is usually part of routine patient evaluation in health care facilities. However, people without a regular source of health care are less likely to be screened for chronic disease and are more likely to use emergency care as a source of health care (11,12). In the prehospital care-delivery system, emergency medical technicians (EMTs) routinely collect medical information that may indicate the presence of chronic disease. EMTs obtain clinical measurements, including blood pressure, on most of the patients they treat in response to a 9-1-1 emergency medical services (EMS) call, regardless of the reason for the call. These data are usually archived without future follow-up and are not used to provide feedback to the patients.

Because EMS is available in most communities, people who have uncontrolled HBP could be identified by these first responders and referred to treatment. Thousands of people receive EMS every year (13); many have undiagnosed or uncontrolled chronic diseases (such as high blood sugar or HBP) that put them at risk for life-threatening events. Many patients whom EMS treat are not transported to a hospital and therefore may not see another health care provider until a more serious event occurs (13).

The objective of our study was to develop an intervention that would provide community residents identified by EMS as having uncontrolled HBP (systolic blood pressure ≥160 mm Hg or diastolic blood pressure ≥100 mm Hg) with information on their medical condition, to offer residents follow-up blood pressure screening, and to determine if a partnership between researchers at the Health Marketing Research Center at the University of Washington, Public Health Seattle King County EMS division, and several large fire departments was an effective way to construct and implement this intervention. As part of our formative research, which aids in identifying target audiences, effective communication strategies, and feasibility of implementation strategies, we developed an intervention designed to report back to EMS patients their medical information (blood pressure values) obtained by EMTs during a response to a 9-1-1 EMS call and to facilitate additional blood pressure screening.

## Methods

The study consisted of 3 parts: 1) informal planning with EMS and other fire service personnel, 2) conducting telephone surveys of community residents who were identified by 9-1-1 EMS responders as being at risk for uncontrolled HBP to assess attitudes and beliefs, and 3) developing an intervention for reaching residents with information about controlling HBP. This study was reviewed and approved by the Human Subjects Division of the University of Washington.

### Study setting

We conducted our study in a large metropolitan area in the Pacific Northwest. We partnered with 4 fire departments in King County, Washington, that served a combined population of 403,260. The smallest department had a population of 49,167 and the largest, a population of 133,543 (15). Together these 4 departments served approximately one-fifth of the King County population.

### Engagement of emergency medical services as community partners

To develop an intervention that was both feasible in an acute care delivery system and acceptable to EMS providers, we conducted informal discussions with EMS personnel at all levels. The medical director of the EMS division of the county health department and study staff met with the fire chiefs of the 4 participating fire departments to discuss how the fire departments' EMS division and the fire departments could collaborate to develop the intervention. Study staff then met with the 4 EMT trainers of the participating fire departments and with individual EMTs and administrative staff.

We did not use a standardized questionnaire in our discussions; however, discussion objectives were the same for each encounter: to assess the feasibility of providing free blood pressure checks at all fire stations, to assess the best intervention delivery method (ie, by EMTs at-scene vs mailing), to stress the importance of complete patient contact information on the medical incident report forms for use in surveys and intervention evaluation, to agree on the proper inclusion criteria for the study, to determine what feedback EMTs would like to receive throughout the process, and to obtain the advice of EMS personnel about the intervention print materials. Study staff used notes of the discussions to design the intervention and develop study strategies. To better understand the daily activities of EMTs, the study coordinator rode with an EMS crew for 1 day.

### Defining the target audience

To define the target audience for the study, we conducted a telephone survey over 3 months with 282 people residing in the areas served by the 4 participating fire departments who had had very high HBP values (systolic blood pressure ≥160 mm Hg or diastolic blood pressure ≥100 mm Hg) during a non–life-threatening 9-1-1 EMS event. The objective of this survey was to assess prior history of HBP, beliefs as indicated by the Health Belief Model (perceived susceptibility, severity, barriers, and benefits), and attitudes toward fire stations as a place for having blood pressure checked.

### Survey instrument

Survey questions (available upon request) assessed demographics; questions based on the Health Belief Model, such as risk perceptions regarding HBP; perceived benefits and barriers to blood pressure control; current blood pressure–control behaviors; and attitudes, and beliefs about 9-1-1 EMS care providers and delivery systems. Data were analyzed with SPSS version 18 (IBM, Chicago, Illinois) by using descriptive statistics (χ ^2^ for frequencies and the Mann-Whitney U test for means). 

### Discussions with emergency medical service partners

As a result of informal discussions with the medical director, fire chiefs, EMT trainers, and EMTs, we designed an intervention that consisted of using medical incident reports to identify eligible participants, training EMTs to complete patient contact information on all medical incident reports, advising 9-1-1 EMS patients of their uncontrolled HBP, and offering free blood pressure checks at many fire stations.

On the basis of our discussions, we decided to use medical incident reports from 4 large fire departments in King County, Washington, to identify eligible participants. EMS personnel fill out a report for each visit, which includes the reason for the 9-1-1 EMS call, diagnosis and procedures EMTs perform, vital signs, patient contact information, whether the patient was transported from the scene, and, if so, by what level of transport, basic or advanced. People receiving EMS were eligible for our study if they were seen during our study period by EMTs in a participating fire district during a 9-1-1 EMS response and had a recorded systolic blood pressure at or higher than 160 mm Hg or diastolic blood pressure at or higher than 100 mm Hg. These values were considered conservative enough to accommodate the "white coat" phenomenon, the tendency of blood pressure to rise during a medical visit (16).

Exclusion criteria were transport to hospital by paramedics rather than EMTs, indicating a more serious medical emergency; being a resident of a nursing home, and thus presumed to have regular access to nursing care with routine blood pressure monitoring and control; being incarcerated or transported by law enforcement agencies; or having a cognitive impairment documented on the 9-1-1 EMS call center dispatch report. Participants had to be aged 18 years and older, have an address in or near 1 of the 4 fire department districts included in our study, and have a home phone number on the medical incident  report or a publicly available residential telephone number.

To conduct our study, we needed patient contact information to identify eligible participants and to evaluate the intervention. Patient contact information was not required on the medical incident report form and required extra effort to obtain on the part of EMTs. In return for this extra effort, we committed to providing regular feedback on community residents' health outcomes to the fire departments. EMT trainers committed to communicate the importance of noting patient contact information on the EMS reports. Our study staff agreed to report regularly on progress and results of the intervention; we sent monthly progress reports on each fire department's success in recording phone numbers and encouraging blood pressure screening.

Initially, we planned for EMTs to advise patients at the scene of their uncontrolled HBP, that is, during the 9-1-1 EMS event. However, in our discussions with EMTs, we determined that an at-scene intervention could interfere with emergency clinical care. Therefore, we decided to mail health information to people after the 9-1-1 EMS visit to reach them at a time when they were not in a stressful emergency situation. EMS personnel suggested we personalize the mailings with pictures of personnel from the EMS serving the person's area rather than using a generic photograph of an EMS crew. We therefore developed 4 direct mail brochures, each with pictures of EMTs from the 4 fire departments.

Because we did not want lack of access to health care to be a barrier to blood pressure screening, we asked that fire stations in the 4 fire departments provide screening free of charge to community members. Although free screening was already a practice in some departments, it was not well established in others, and we negotiated free blood pressure checks at many (25 out of the 30) fire stations in participating departments. We decided it would be too complicated to ask fire personnel to write down contact information for each person coming to their fire station for a blood pressure check, so we evaluated this behavior via telephone survey rather than extensive logs at fire stations.

## Results

We contacted 794 people whom EMTs had identified as at risk for uncontrolled HBP for our interviews. Of the 794 interviews attempted, 282 (35.5%) community residents began the survey, and 270 (34%) finished it; 12 persons began the survey but dropped out during the interview. The denominator for individual questions is based on the number who answered that question, and thus the denominators vary slightly because of the drop-outs, those who declined to answer a particular question, and those who answered a question as "unknown" or "do not remember." Age and sex were available for all participants from the information recorded by EMTs on their incident reports, if not answered on the survey.

Demographic information included age, sex, marital status, health insurance status, race/ethnicity, and educational attainment. Sixty-six percent (n = 186/282) of participants were women. The mean (standard deviation [SD]) age was 64.1 (16.6) (n = 282); 70% (n = 173/245) had more than a high school diploma, and 86% (n = 204/238) were white. These demographics helped us develop a target profile of an older, white, fairly well educated, mostly female study population.

Of the 512 who were not interviewed, 28% (n = 144) refused, 22% (n = 111) had a busy telephone line or answering machine, 18% (n = 92) had incorrect or disconnected telephone numbers, 14% (n = 71) were unable to participate because of a disability, 8% (n = 41) were unable to participate because of a language barrier, approximately 2% (n = 11) were deceased, approximately 1% (n = 7) were hospitalized, and 7% (n = 35) were "other." Selected variables were recorded as part of the medical incident reports and were available to compare the 282 participants who began the survey with the 512 who were not interviewed: age, sex, location of the 9-1-1 EMS visit (home vs away from home), and whether the patients were transported from the scene for further care. The 282 subjects were found to be representative of the 512 for whom an interview was attempted unsuccessfully on all comparisons except age; the 282 were younger (64.1 y; SD, 16.6 y) than the 512 participants who were not interviewed (66.5 y; SD, 18.9 y).

Most (67%, n = 180/269) respondents reported a history of hypertension, were medicated for HBP (67%, n = 180/270), and believed HBP to be a serious health threat (78%, n = 201/259). Nearly all participants (95%, n = 246/259) believed that regular screening was important for personal health. On the basis of this information, we determined our target audience would likely be mostly undercontrolled hypertensive patients, as all these patients were selected for the interview on the basis of extremely high blood pressure values in the medical incident reports. However, a significant proportion, about one-third, might be at risk for being newly diagnosed patients as they had not received a diagnosis by a health care provider but had extremely high blood pressure values during a recent 9-1-1 visit.

Because we wanted to improve access to screening for all patients, particularly those without regular access to care, we asked about patients' familiarity and comfort with local fire stations as a place for follow-up blood pressure screening. Almost every survey participant (95%, n = 249/262) knew of a nearby fire station and reported it would be easy to go there (88%, n = 226/257). Forty-four percent (n = 115/262) reported ever having visited a local fire station. Sixty-five percent (n = 138/211) reported that they believe a fire station would be a good place to learn about health, and most (82%, n =136/165) reported they would feel comfortable having their blood pressure checked at a local fire station. Furthermore, 96% (n = 239/250) of participants viewed EMTs as competent to evaluate and treat medical problems. Sixty-five substantial (n = 166/254) strongly agreed they would trust EMTs to evaluate and treat their medical problems.

### Development of intervention strategy and materials

On the basis of survey results and discussions with EMS personnel, we determined that a partnership may be feasible between researchers at an academic institution, the EMS division of the local health department, and 4 fire departments to increase community awareness of uncontrolled HBP. Data showed that the health beliefs as posited in the Health Belief Model were held strongly in our target audience. Furthermore, EMTs were perceived as credible spokespeople, and the majority of participants reported that they would feel comfortable visiting a fire station for a blood pressure check. On the basis of our research, we developed an intervention for reaching people with uncontrolled HBP identified during a 9-1-1 EMS event. On the basis of conversations with EMS providers, the intervention will be conducted via direct mail. We developed a brochure mailer (Figure) that notifies the recipient that their EMS crew is concerned about a HBP reading taken at the time of a 9-1-1 EMS visit. On half the brochures that will be mailed to residents the actual blood pressure values of the patient are listed in an attempt to further increase perceived susceptibility to uncontrolled HBP. As HBP was already seen as a serious health threat, we decided not to add more content around severity of HBP. We did, however, include information on the benefits of blood pressure monitoring and treatment. To reduce barriers to screening we included a list of local fire stations where blood pressure can be checked.

**Figure.**
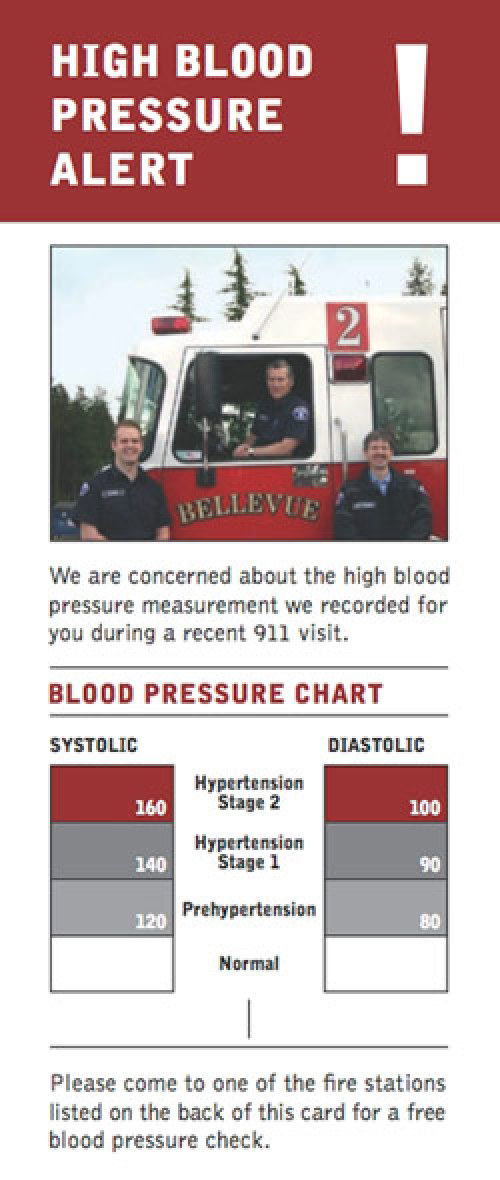
Mailer sent to recipients of emergency medical services who had a recorded systolic blood pressure of ≥160 mm Hg or diastolic blood pressure of ≥100 mm Hg. The mailer is the size of a business envelope and includes a list of participating fire stations on the reverse side. On half the brochures that will be mailed to residents, the actual blood pressure values of the patient will be listed in an attempt to further increase perceived susceptibility to uncontrolled HBP. The brochure also includes information on the benefits of blood pressure monitoring and treatment.

## Discussion

Our intervention strategy has been tested and shown to be successful in the past in a similar setting. A direct-mail campaign to urge people to call 9-1-1 EMS for symptoms of myocardial infarction resulted in a significant increase in calls for chest pain among patients with a history of heart disease (17). A commercial mailing list that consisted of names and addresses only was used in that study; therefore, information on recipients' medical history was not available before the implementation of the intervention. Using medical incident reports instead of a commercial mailing list will allow us to identify and target at-risk community residents.

There are limitations to our study. The intervention developed and reported here was designed for a specific community. Fire departments in King County, Washington, have a history of participation in patient and community outreach (18,19) and were excited about the project. Therefore, results may not be applicable to communities with different EMS systems or a different population. However, the process by which we developed our intervention should be applicable to other communities.

The intervention we developed will give participants health information they may not have known about, on which they may choose to act. In addition, patients will be given the opportunity to receive a free blood pressure check at a local fire station, which will allow access to screening for all residents of our community.

Our study showed that a partnership is feasible between researchers at the Health Marketing Research Center at the University of Washington, Public Health Seattle King County EMS division, and community EMS and that such a partnership can provide community residents with information on their health and health risk factors that may not be otherwise available to them. Fire stations are conveniently located in most neighborhoods, and fire station EMS personnel can serve a useful role in providing blood pressure screening to their community.
